# Telemedicine consultation for emergency patients’ attention: a clinical experience from a high complex university hospital from Latin America

**DOI:** 10.1186/s12913-023-09520-6

**Published:** 2023-05-30

**Authors:** Laura Libreros-Peña, Jaime A. Quintero, Arnold Gelves, Juliana Alarcón, Sergio Morales, María Fernanda Escobar, Andres M. Valencia, Sara Guzmán, Julio Diez-Sepulveda

**Affiliations:** 1 Centro de Investigaciones Clínicas (CIC), Fundación Valle del Lili, Carrera 98 No.18-49, Cali, 760032 Colombia; 2 Unidad de alta complejidad Obstétrica, Fundación Valle del Lili, Cali, Colombia; 3 Departamento Medicina de Emergencias, Fundación Valle del Lili, Cali, Colombia; 4 Departamento de posgrados - Medicina de Emergencias, Facultad de Salud, Universidad Icesi, Cali, Colombia; 5 Departamento de Ginecología y Obstetricia, Facultad de Salud, Universidad Icesi, Cali, Colombia; 6 Facultad de Ciencias de la Salud, Universidad Icesi, Cali, Colombia

**Keywords:** Telemedicine, Implementation, Telehealth, Emergency

## Abstract

**Introduction:**

As a result of the new coronavirus pandemic, a highly complex academic hospital in Latin America implemented a telemedicine service for the care of obstetric, pediatric, and adult patients. In 2020, regional emergency services collapsed due to the increase in demand for care, generating the need to open expansion services and seek strategies to provide timely care to consulting patients.

**Objective:**

We retrospectively describe the clinical experience of patients who consulted the emergency department via telemedicine across a videoconference tool using digital platforms.

**Methods:**

A descriptive study with retrospective data collection was conducted to describe the implementation of the teleconsultation care model for patients. We constructed the clinical process indicators to evaluate the model.

**Results:**

A total of 4652 teleconsultations were registered. Telemedicine consultation was above 50% in the country and department and above 90% in Cali city. The average waiting time for care was estimated to be 1:59:52 h. A total of 275 patients were transferred to the emergency room after consultation. The principal reasons for consultation in the institutional telemedicine program were respiratory and gastrointestinal symptoms. Teleconsultations related to SARS-COV 2 infections reported 3775 patients (3127 with unidentified virus and 648 with the identified virus).

**Conclusions:**

Telemedicine is a tool that provides support and guidance to patients who consult emergency departments, reducing barriers to access health care and decreasing emergency department collapse.

## Introduction

Telemedicine is defined as the provision of health care remotely via information and communications technology [1] and has been proposed during the last two decades as a tool with potential benefits for health care [2, 3].

During the 2020 COVID-19 outbreak, health systems worldwide experienced an unprecedented collapse, generating accelerated growth in the articulation of alternative care modalities such as telehealth and electronic health seeking to provide health care using technologies to exchange information in the diagnosis, treatment, prevention, and clinical surveillance of diseases [3, 4].

In Colombia, the implementation of information technologies and telehealth outreach began in 2002, and it was a broad regulatory framework that established operating and enabling conditions [5, 6]. However, due to the COVID-19 pandemic, the use of telehealth was stimulated by the Ministry of Health to guarantee access to comprehensive health throughout the national territory [7].

During the COVID-19 pandemic outbreak, Fundación Valle del Lili (FVL) University Hospital and other institutions worldwide experienced an unprecedented need to develop strategies to provide medical care to many patients during confinement measures to reduce the collapse of the emergency department.

“Siempre” is an outpatient teleconsultation care model implemented in Fundación Valle del Lili, Latin American University Hospital in March 2020. We aim to describe the clinical experience using telemedicine for emergency department consultation and demonstrate the behavior of some attention indicators.

## Methods

### Design and context

Fundación Valle del Lili (FVL) is a high-complexity university hospital and urban medical center located in the southwestern region of Colombia. It has been constituted as a reference center for Latin America due to the volume of patients and experience in health services for complex diseases. Annually, it provides care to approximately 12,000 patients from all over the country, mainly from the southwestern region. It is located in Santiago de Cali, one of the three main capitals of Colombia.

The ethics committee in biomedical research at University Hospital (Comité de Ética en Investigación Biomédica de la Fundación Valle del Lili)) approved this study (Register No. 09-2021, approved on March 24, 2021, Institutional act No. 06 of March 24, 2021, protocol number 1742). This study applying the declaration of Helsinki Ethical Principles for Medical Research Involving Human Subjects. This study adhered to the standards of the STROBE guidelines and not required an informed consent due to the research design (resolution No. 008430 of 1993, article 11, numeral A of the Ministry of Health and Social Protection of Colombia).

### Patients

All patients older than 18 years treated in the emergency department from April 2020 to February 2022 were included. Patients who required taking vital signs and face-to-face physical examination for clinical decision-making, pregnant women, and pediatric patients were excluded. Demographics and clinical variables were collected. The data were recorded in the institutional clinical record system (SAP).

### Overview of the siempre teleconsultation program

Telehealth was implemented in Colombia in 2002 with a broad regulatory framework. Resolution 1448 of 2006 established the Enabling Conditions for the Telemedicine modality (applicable in situations in regions with limitations of offer or access to the health service from an institution classified as a referral or reference center). In 2010, Law 1419 of 2010 [8] established the concepts of Telehealth, Telemedicine, and Tele-education.

Subsequently, resolutions or laws were issued that modified or provided additional data (Law 1438 of 2011 [9], Law 1450 of 2011 [9], and Resolutions 1441 of 2013 [9]). In 2019, resolution 2654 established the provisions for telehealth and parameters for the practice of telemedicine [10]. Resolution 3100 of November 2019 specified the rules in telemedicine for each specialty.

Telemedicine has been included in the benefit plans of the subsidized and contributory systems since 2019.

“Siempre” is a telemedicine program implemented emergently as an institutional response measure due to the health emergency coronavirus pandemic. The patient or caregiver initiates a call with an institutional agent who answers, defines the type of patient insurance, establishes the age, and verified identification data by taking a photo of the identification document using the platform Microsoft Teams®. After the appointment is assigned, the agent sends the consent form and authorization to process personal data.

**Fig. 1:**
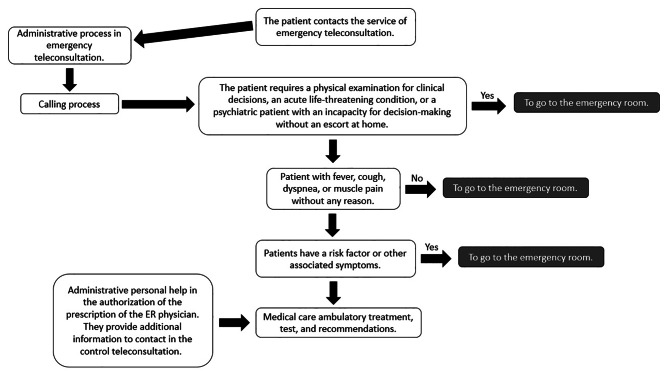
Quality and Clinical Indicators FVL

After these steps, an emergency physician interviews the patient and provides attention, registering clinical history, generation of orders, and medical formulation through SAP (Fig. 1). Once the consultation is finished, a PDF file is sent to the patient’s previously provided email and authorized for contact.

### Indicators

**Fig. 2:**
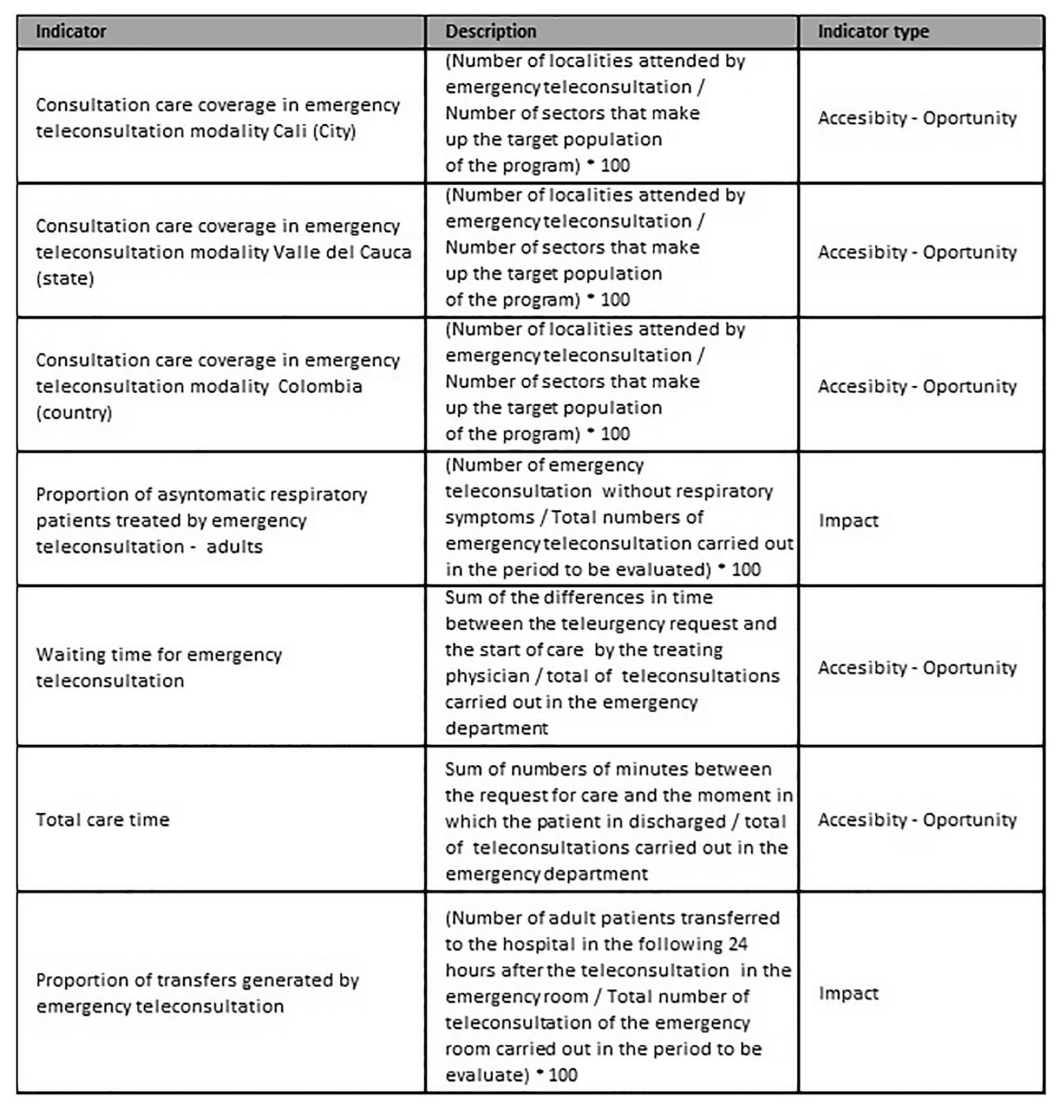
Emergency Telemedicine Flowchart

Quality and clinical indicators were set previously to evaluate the model. Quality indicators seek to evaluate local and national coverage, the proportion of patients attended via telemedicine for emergency causes related and not related to COVID-19, average attention times, and clinical indicators were set to identify clinical outcomes related to the subsequent attention in the telemedicine program (Fig. 2. **Quality and Clinical Indicators FVL**). The quantitative variables were expressed through the measure of central tendency and dispersion. Categorical variables were described in proportions.

## Results

Between April 2020 and February 2022, a total of 4652 teleconsultations were performed. The mean age was 37 years old. Females represented 62.1% of teleconsultations. The most common age group was young (74%). Coverage in telemedicine was above 50% in the country and the department of Valle del Cauca but above 90% in Cali city (Table 1).

**Table 1:** Demographic characteristics

Variables	Total, n: 4652 (%)
Gender	
Female	2889 (62.1)
Male	1763 (37.9)
Age*	37 (29–49)
**Categories of Age Groups**	
Young (14–26 years)	3441 (74)
Adult (27–59 years)	737 (15.8)
Old Adult (≥ 60 years)	474 (10.2)
**Emergency teleconsultation coverage**	
Cali, (Neighborhoods, n:22)	20 (91)
Valle del cauca, (Cities, n:42)	23 (55)
Colombian (Departaments, n:32)	19 (59)

** Mean, interquartile range

**Fig. 3:**
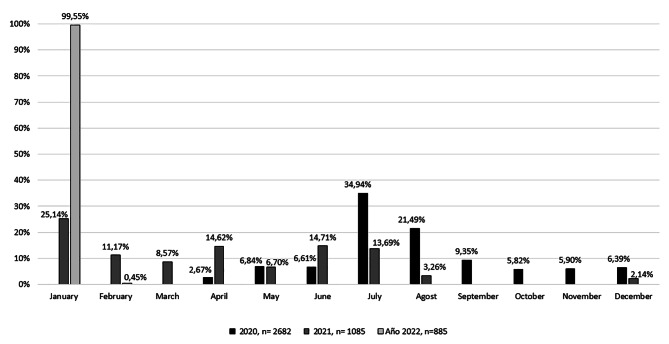
Monthly distribution of emergency Teleconsultations between 2020 and 2022

In 2020, the monthly distribution of emergency teleconsultations was significantly higher between July and August. Between September and November, teleconsultation was not used by patients. In 2021, January (25.14%) was the month with the most teleconsultation, followed by June (14.71%) and April (14.62%) (Fig. 3).

According to the preset quality indicator measures, the average waiting time (time between the administrative process of the teleconsultation and the medical interview) was estimated to be 1:59:52 h, and the total attention time was approximately 3:14:02 h.

**Fig. 4:**
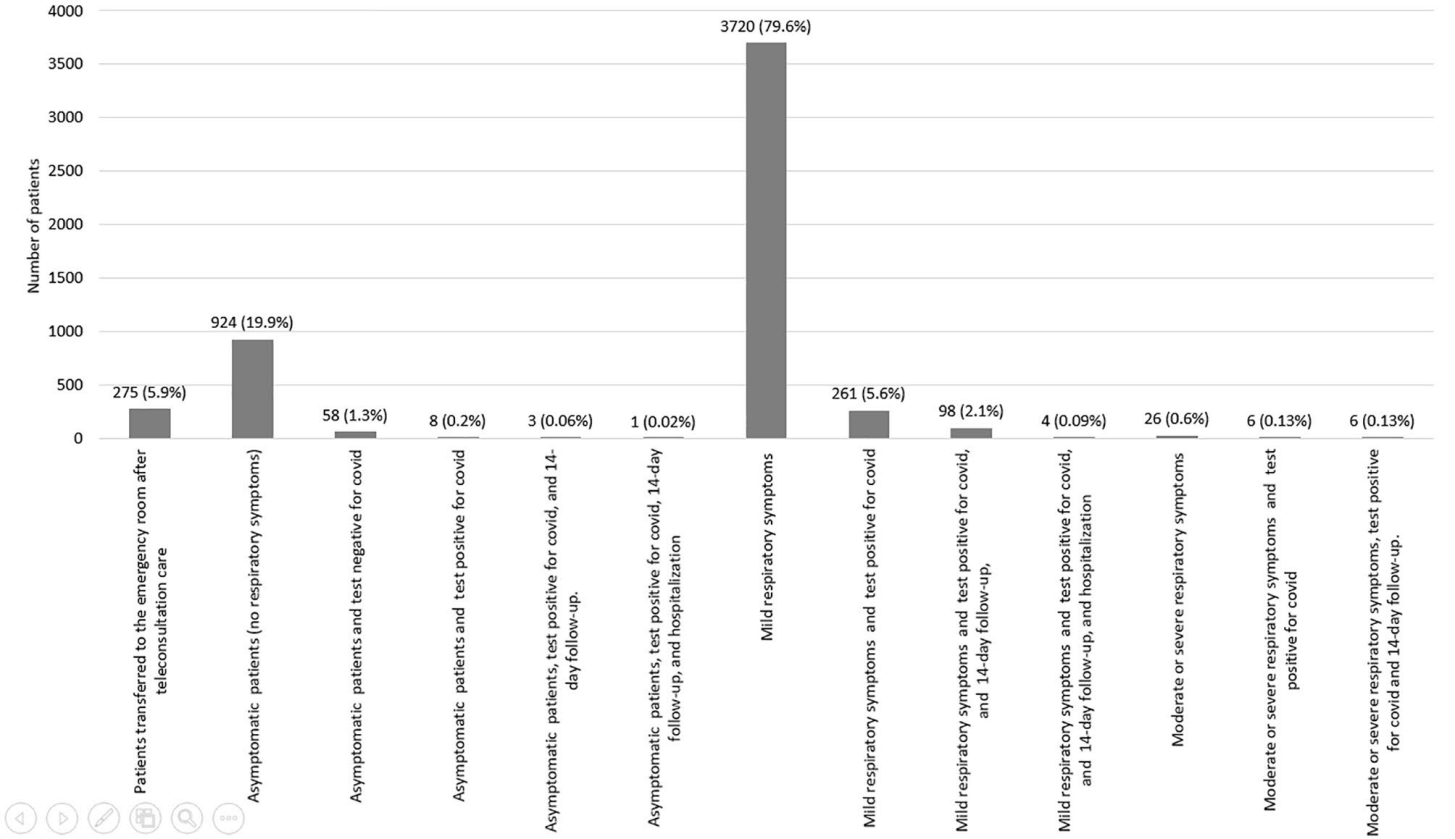
Respiratory Symptoms Consultations

In total, 275 patients were transferred to the emergency room after teleconsultation. The most common cause of consultation was related to respiratory symptoms in 78.9%, and only 7% of these patients presented a positive test for COVID-19. Follow-up for 14 days was realized in 2% (n: 98). Only 4%(n: 4/98) were hospitalized. In addition, 26 patients presented severe or moderate respiratory symptoms. Only 23% (n: 6/26) of patients had a positive COVID-19 test result and were hospitalized (Fig. 4).

Table 2 summarizes the most prevalent diagnoses of care by teleconsultation. The principal reasons for consultation in the institutional telemedicine program were respiratory symptoms. Teleconsultations related to SARS-COV 2 infections reported 3775 patients. However, a total of 648 patients with the virus were identified.

**Table 2:** Causes of tele-emergency care

Dx	Diagnosis	Total
**U072**	COVID-19 (unidentified virus)	3127
**U071**	COVID-19 (identified virus)	648
**J069**	Upper Respiratory tract infection	402
**B349**	Viral infection, unspecified	297
**Z208**	Contact with and (suspected) exposure to other communicable diseases	175
**A09X**	Infectious gastroenteritis and colitis, unspecified	154
**J00X**	Acute nasopharyngitis (common cold)	131
**I10X**	Hypertension	112
**R51X**	Headache	99
**J22X**	Acute lower respiratory infection, unspecified	76

## Discussion

This study describes the experience in the implementation of the telemedicine program during the outbreak of covid 19 in our hospital. We observed great coverage in the access and optimization of the opportunity and use of service in health care. The current pandemic caused by COVID-19 caused a health emergency that collapsed health systems globally. In our study, the increase in the number of consultations in emergency rooms at all levels of care was evident. The implementation of emergency teleconsultation in our hospital contributed to a decrease in the number of face-to-face consultations without affecting the humanization and quality of care.

The coverage objective before the implementation of the emergency teleconsultation was pre-established at 10% in the city of Cali. However, one of the most relevant findings in our study was the coverage because above 50% of the country and 90% of our city. It contributed to providing medical care. In addition, we avoided 4,377 face-to-face consultations limiting the risk of exposure and maximizing the protection of health personnel. In a medium-complexity hospital in North Texas (USA) that receives approximately 50,000 emergency consultations annually, less than half of the consultations registered in recent years in our hospital. Patients with severe or moderate respiratory symptoms were hospitalized immediately and patients with mild symptoms were evaluated by telecare. Over two weeks, 500 patients were registered, 140 (28%) with a high risk of infection by COVID-19, and only 30% of the patients presented hemodynamic instability with an indication for emergent management [11].

With the start of the pandemic, countries such as India regulated the use of telemedicine early, not only to assume care coverage concerning the number of patients with COVID-19-related symptoms but also to regulate the care of chronic patients subsequently during this period; several nations chose these public health policies, including Latin American seeking to benefit all population groups and care of different specialties [12].

In a descriptive study carried out in two centers for weight management located in New York and San Diego in the United States, it was documented that there is a large number of patients who do not attend their periodic medical check-ups for different reasons, such as traveling long distances and being absent from work. The study suggested that telemedicine eliminates these barriers for the patient, affecting health system costs derived from medical care bilaterally [13]. Additionally, adherence to follow-up through the virtual modality and fewer contagions were elucidated [12].

The COVID pandemic decreased the Colombian economy by 6.8% and at least 8 points in the national unemployment rate to almost 11.5 in the 13 principal cities in 2020 [14]. The Pacific region saw exacerbated social problems, presenting a marked deterioration in its growth indicators, and departments such as Valle del Cauca increased their monetary poverty levels by 10% points in 2020. This impact generated a detriment to the health system and food safety. In terms of health, the Pacific region 2020 presents a significant lag in compliance with Sustainable Development Goals (SDG) 3 in most of the territory, according to the measurement of compliance with the SDG at the country level carried out by Propacifico. The mandatory isolation decreed in Colombia allowed medical centers of the public and private network to prepare by increasing installed capacity, highly trained human talent, and biosecurity measures to reduce the contagion and spread of the virus.

Teleconsultation had not been implemented in our country. Its implementation has been a very useful alternative, which made it possible to adequately respond to the great demand for medical care related to the current pandemic in emergency rooms and the follow-up of patients with chronic pathologies in the outpatient clinic. There was even evidence of a significant increase in national, regional, and local coverage [15].

The implementation of information technologies in Colombia began in 2002. Resolution 2654 of 2019 established the provisions for telehealth and the parameters of the telemedicine practice: Tele-education, Tele-orientation (to direct and provide information to users about health conditions), Tele-support (support between the human talent of health), and four areas of telemedicine [16]. Before the SARS-COV-2 pandemic, private health institutions provided 19,341 consultations in telemedicine, teleconsultation, or teleguidance [16]. In 2020, there was a 192% growth in the services offered through the telemedicine modality [17]. There are no records or studies of the effect of telemedicine on emergency attention in medical care in Colombia and Latin America during the outbreak.

Four waves were reported in Colombia: The first wave was between March and October 2020, the second wave was between October 2020 and March 2021, the third wave was between March and June 2021, and the fourth wave was between December 2021 and February 2022. During the waves, the number of patients consulted became evident. However, the fourth wave reported a high number of teleconsultations in January. This result may be more related to the knowledge of the use of teleconsultation and the experience of the opportunity for attention compared to the face-to-face consultation.

In our study, respiratory symptoms related to covid were the most common cause of emergency teleconsultations. A study performed in North Carolina (USA) described the patterns in virtual consultations by symptoms of COVID-19. A total of 733 virtual consultations were performed over two months. A total of 257 patients (35.1%) were related COVID-19-like symptoms, and 92 patients (12.5%) were confirmed cases [18]. In our study, 0.8% of patients with no respiratory symptoms had positive COVID tests, and 7% of patients with mild respiratory symptoms had positive COVID tests. This result could be explained by the panic generated in the population due to the relationship of any common flu symptom with a case of covid.

ER physicians were instructed in the teleconsultation care model and performed the anamnesis to prescribe clinical management with more criteria and resolutive capacity than a general physician (Fig. 2). Only 5.9% were referred to the emergency room minimizing the risk of infection. This strategy decreases the number of face-to-face visits and controlled collapse in the emergency room with good use of human and hospital resources.

Women and young people were the most frequent group of people who used teleconsultation. Several data sources suggest that women make higher use on average of primary care than men. This result is similar to the report from OMS and OPS [19, 20]. Young people were the most common age of group teleconsultation. This group has more opportunities to use due to access to and knowledge of the technology [21–24].

All patients received guidance and pharmacological and nonpharmacological management plans, general recommendations, warning signs, and consultations. In addition to follow-up in the following days and weeks, with the aim of evaluating the improvement of symptoms, the presence of warning signs, and control with the results of laboratory tests. Our work group must carry out a timely follow-up of each patient, according to their particular needs, for example, knowledge of diagnostic tests and clinical evolution. This work required the joint effort of patient satisfaction and health professionals.

Patients accepted the telemedicine program well, and high satisfaction was also perceived. A descriptive study on patients with osteoporosis described the care experience through telecare. Most of them expressed their comfort with using new information technologies and recognized the same quality. In addition, the reduction in trips and costs was relevant. However, this study lacked further follow-up [13]. It can be concluded from previous experience that telemedicine offers similar results to face-to-face clinical scenarios [25]. During the pandemic, the use of telemedicine was not exclusively in the care of patients with chronic pathologies on a routine basis; coverage in patients with medical emergencies was another advantage.

This report highlights the need to continue innovating in health technology to solve problems in public health and social impact. Our experience in innovating technology for the benefit of patient care in southwestern Colombia can support this process from the evidence.

### Limitations and strengths

The retrospective nature confers the main limitation of this study. Although guidelines and strategies have been proposed to promote the implementation of telemedicine services in Colombia, this is thus far the only study that has been conducted in the country and one of the few in Latin America describing the implementation of this type of care model, and we cannot compare our results and experience with other institutions of the region.

More research is needed to show that telemedicine improves clinical results in the patient, which can favor the establishment of care guidelines with characteristics in standardized provision [26]. In the outpatient and emergency setting, in addition to primary health care programs, they can be implemented in larger populations, all without leading to excessive use of telemedicine.

### Future implications

The use of technology facilitates the remote access of patients to quality health services. Its implementation in the surveillance and follow-up of patients in primary health care models has shown relevant results [27], allowing increased access to care and impacting the costs associated with the provision of health services [28]. During the time of implementation of the telemedicine model, its great benefits in the care of patients with acute and chronic exacerbated pathologies were evident, and the patients presented high satisfaction with acceptable safety outcomes and favorable clinical evolution. Now, the challenge is to continue building an efficient and high-quality telemedicine service. Increasing the opportunity in the consultation and the number of available specialists will guarantee the continued implementation of this care model. The telemedicine consultation taught us that technology is a tool that allows us to positively impact our patients through a communication device. Patient education, symptom relief, and support during the illness characterized the efforts of our medical team.

The application of telemedicine in different areas of health entails not only clinical challenges but also other technological, legal, ethical, and social aspects. Ease of access, adequate connectivity, and user capacity must be taken into account. Many challenges exist related to technology interoperability, the digital divide, and usability. Importantly, evidence is needed to support this paradigm shift in care delivery to ensure the quality and efficacy of care delivered via telemedicine [29].

## Conclusions

Teleconsultation can be a useful tool for providing medical assistance and lowering barriers to access to health providers, which are common in developing countries. This teleconsultation model allowed the continuity of comprehensive attention in a local, regional, and national territory. However, it does not replace face-to-face care for different reasons. It can be a tool that allows first contact and accompaniment of patients, improving the occupation in the emergency room.

## Data Availability

The data that support the findings of this study are available from Fundación Valle del Lili but restrictions apply to the availability of these data, which were used under license for the current study, and so are not publicly available. However, data are available from the corresponding authors upon request reasonable and with permission from the hospital.
